# Elevated Immunoglobulin G as a Predictor of Progression to Severe Lung Disease in Cystic Fibrosis: A Longitudinal Cohort Study

**DOI:** 10.3390/jcm14124331

**Published:** 2025-06-18

**Authors:** Ori Goldberg, Siwar Shekh-Yusuf, Miri Dotan, Moshe Heching, Eyal Jacobi, Meir Mei-Zahav, Hannah Blau, Huda Mussaffi, Dario Prais

**Affiliations:** 1 Pulmonary Institute, Schneider Children’s Medical Center of Israel, Petach Tikva 4920235, Israelmeir_zahav@clalit.org.il (M.M.-Z.);; 2 School of Medicine, Faculty of Medical and Health Sciences, Tel Aviv University, Tel Aviv 6997801, Israel; 3 Pediatric Pulmonary Service, Kaplan Medical Centre, Rehovot and the Faculty of Medicine, Hebrew University of Jerusalem, Jerusalem 7661041, Israel; 4 Pulmonary Institute, Rabin Medical Center, Petah Tikva 4941492, Israel

**Keywords:** cystic fibrosis, immunoglobulins, lung transplant

## Abstract

**Background:** Elevated immunoglobulin G (IgG) levels are associated with worse lung function and disease severity in people with cystic fibrosis (PwCF). This study evaluated whether elevated IgG levels—defined as values above the 97.5th percentile (Z-score ≥ 1.96 standard deviations above the mean)—can predict progression to severe lung disease. **Methods:** A retrospective cohort study of children and adults with CF at a single-center clinic was performed. Patients with elevated baseline IgG Z-scores were compared to those with normal or low IgG levels. Progression to severe lung disease was defined as % predicted FEV_1_ < 40%, referral for lung transplantation, or death. Kaplan–Meier survival curves and Cox models were used to analyze clinical outcomes. A sensitivity analysis was conducted for patients aged 18 years or older. **Results:** Of 97 patients, 31 (31.9%) had elevated IgG levels. Progression to severe lung disease occurred in 14 (14.4%) patients, 12 (85.7%) of whom had elevated IgG. These patients were significantly older and had a higher prevalence of Pseudomonas aeruginosa colonization. Among adults, those with elevated IgG had lower baseline % predicted FEV1 and greater annual lung function decline. Elevated IgG was independently associated with progression to severe lung disease (adjusted hazard ratio [aHR]: 9.8; 95% CI: 1.9–48.6), even after adjusting for Pseudomonas colonization and annual % predicted FEV1 decline. **Conclusions:** Elevated IgG was associated with progression to severe lung disease in PwCF and correlated with older age, Pseudomonas colonization, and—in adults—lower baseline lung function and faster decline. These findings highlight elevated serum IgG as a meaningful prognostic biomarker for identifying high-risk PwCF who may benefit from closer monitoring and earlier intervention.

## 1. Introduction

Cystic fibrosis (CF) is an inherited, life-threatening, multisystem disease primarily involving the lungs and the gastrointestinal tract [1]. Mutations in the CFTR gene (cystic fibrosis transmembrane conductance regulator) impair the production or function of the CFTR protein, leading to dysregulation of sodium and chloride ion transport across epithelial cells in multiple organs [2].

Inflammation plays a pivotal role in CF lung disease. Chronic infections, particularly with Pseudomonas aeruginosa, provide a persistent stimulus to the immune system, driving a cycle of inflammation and tissue damage [3]. Additionally, defects in CFTR itself may contribute to increased production of inflammatory mediators, such as IL-8, which promotes neutrophil migration to the airways [4]. This initiates a vicious cycle of inflammation, characterized by the release of additional mediators, structural damage to the airways, bronchiectasis, fibrosis, and, ultimately, respiratory failure [3].

Over the past few decades, elevated levels of immunoglobulins, particularly immunoglobulin G (IgG), have been associated with severe lung disease in CF [5,6]. Most studies investigating this relationship have been cross-sectional or with limited clinical follow-up. A longitudinal study conducted a decade ago demonstrated that an increase in IgG Z-scores in children with CF correlated inversely with changes in % predicted of forced expiratory volume in 1 s (FEV1pp) over a 4-year follow-up period [7].

Another longitudinal study supported these findings, reporting that higher serum IgG levels were associated with a more rapid decline in FEV1pp among pediatric patients [8].

Recent studies have further elucidated the role of immunoglobulins in CF. Elevated IgG and IgA levels have been observed as indirect markers of worsening pulmonary function tests (PFTs), though they showed no significant relationship with Pseudomonas aeruginosa colonization [9]. Elevated IgG levels have been linked to severe disease parameters, including lower FEV1pp and lower Shwachman-Kulczycki scores [10]. However, there remains a paucity of data on the longitudinal clinical outcomes of PwCF with hypergammaglobulinemia, particularly in broader cohorts that include both pediatric and adult patients.

This study aims to address this knowledge gap by evaluating whether significantly elevated IgG levels—defined as values above the 97.5th percentile (Z-score ≥ 1.96 standard deviation (SD)) at baseline—could serve as a predictor of progression to severe disease in people with cystic fibrosis. Severe progression was defined by key clinical outcomes, including the development of advanced lung disease (FEV1pp < 40% predicted), referral for lung transplantation, or death. By examining the prognostic value of IgG, this study seeks to determine whether this widely available and inexpensive biomarker could be integrated into routine clinical practice to identify high-risk people with cystic fibrosis (PwCF) who may benefit from earlier, targeted interventions and closer monitoring.

## 2. Methods

### 2.1. Study Design and Population

We conducted a retrospective longitudinal cohort study at the Cystic Fibrosis Centers of Schneider Children’s Medical Center and the Pulmonary Institute of Rabin Medical Center, Petah Tikva, Israel. Both centers are university-affiliated tertiary care institutions providing comprehensive care for PwCF across pediatric and adult age groups, respectively. This study included patients diagnosed and followed at these centers between 1996 and June 2020, prior to the widespread implementation of CFTR modulator therapy.

Eligible patients were those with baseline immunoglobulin G (IgG) Z-scores measured 3 to 7 years before their last follow-up, documented FEV1pp, and complete follow-up data. For patients who underwent lung transplantation, data were collected until the date of transplantation. Exclusion criteria included patients younger than five years of age at last follow-up and those with autoimmune diseases or known immunodeficiency disorders.

### 2.2. Data Collection and Variable Definitions

Annual IgG measurements during a routine follow-up were converted into age-adjusted Z-scores using established reference values [10]. IgG levels were assessed during periods of stable clinical status; fluctuations during exacerbations were not evaluated. Patients were classified as having a positive history of elevated IgG if they had an IgG Z-score ≥ 1.96 at baseline.

Baseline characteristics included sex (male/female), age, pancreatic status (sufficient/insufficient, based on enzyme use), and genotype (mutations I–III were classified as severe, while IV–V or unknown/missing mutations were classified as non-severe). Background variables collected at the baseline visit included body mass index (BMI) standard deviation scores (calculated using CDC growth charts; for adults, BMI was calculated using the CDC reference for 19-year-olds), Pseudomonas aeruginosa infection status at baseline (defined as at least one positive sputum culture in the last 12 months), and cystic fibrosis-related diabetes at baseline (as documented in medical records).

### 2.3. Primary Outcome

The primary outcome was progression to severe lung disease and was defined as the occurrence of any of the following outcomes: FEV1pp < 40% predicted, referral for lung transplantation, or death [11].

### 2.4. Statistical Analysis

Descriptive statistics are presented as mean ± standard deviation (SD) for normally distributed continuous variables, median with interquartile range (IQR) for non-normally distributed continuous variables, and frequencies with percentages for categorical variables. Group comparisons for categorical variables were conducted using the chi-square test or Fisher’s exact test when expected cell counts were <5. Continuous variables were compared using the independent samples. Student’s *t*-test for normally distributed data and the Mann–Whitney U test for non-parametric data.

Survival analysis was performed using Kaplan–Meier survival curves and Cox proportional hazards regression models to evaluate the association between elevated baseline IgG levels and the risk of progression to severe disease. Patients entered the survival analysis window between 3 and 7 years prior to their last follow-up, with the event status determined by their outcome at the subsequent follow-up visit. The time between baseline and follow-up assessments was used as the time-to-event variable. The multivariable Cox model was adjusted for potential confounders, including Pseudomonas aeruginosa colonization and annual FEV1pp decline. Hazard ratios (HRs) and their corresponding 95% confidence intervals (CIs) were reported.

A two-tailed *p*-value < 0.05 was considered statistically significant. All analyses were performed using IBM SPSS Statistics (version 25, Chicago, IL, USA).

### 2.5. Ethics Board Approval

This study was approved by the local ethics Institution Review Board (IRB), 0643-20-RMC, on 29 September 2020.

## 3. Results

A total of 138 PwCF over the age of five were initially evaluated. Forty patients were excluded due to insufficient follow-up data on IgG levels, FEV1pp, or loss to follow-up, and one additional patient was excluded due to repeated hypo-IgG measurements. Ultimately, 97 patients met all inclusion criteria. Demographic and clinical data of the cohort are presented in Table 1.

### 3.1. Elevated IgG Levels vs. Normal/Low IgG Group

The study population was categorized into two groups based on their baseline IgG Z-score as follows: 31 patients (32.0%) were classified as having elevated IgG (≥1.96 SD), while 66 patients (68.0%) had normal/low IgG (<1.96 SD). The median follow-up duration was similar between groups at approximately four years. Patients with elevated IgG were significantly older at baseline (median: 25.4 years, IQR: 18.2–36.0) than those in the normal/low IgG group (median: 16.5 years, IQR: 9.75–26.25, *p* = 0.004).

At baseline, patients in the elevated IgG group had a trend toward worse lung function, with a median FEV1pp of 64.0% (IQR: 43.0–77.7), compared to 96.0% (IQR: 81.3–103.5, *p* = 0.06) in the normal/low IgG group. Over time, lung function decline was more pronounced in the elevated IgG group, with a median annualized FEV1pp decline of -1.18% per year (IQR: −3.3 to −0.42), compared to −0.64% per year (IQR: −2.07 to +0.74, *p* = 0.071) in the normal/low IgG group.

Pseudomonas aeruginosa colonization was significantly more prevalent in the elevated IgG group, affecting 67.7% of patients compared to 37.9% in the normal/low IgG group (*p* = 0.006). Among those who progressed to severe disease, 75.0% had Pseudomonas colonization at the four-year mark, compared to 37.3% of those who remained stable (*p* = 0.005). Baseline characteristics, including CFTR mutation severity, sex distribution, pancreatic status, and CF-related diabetes, did not differ significantly between groups (Table 1).

By the end of follow-up, 14 patients (14.4%) met criteria for progression to severe lung disease. This outcome was significantly more frequent in the elevated IgG group, where 12 of 31 patients (38.7%) progressed to severe disease, compared to only 2 of 66 patients (3.0%) in the normal/low IgG group (*p* < 0.001).

### 3.2. Subgroup Analysis: Patients Aged >18 Years

Among the 52 adult patients, 24 (46.2%) were in the elevated IgG group and 28 (53.8%) were in the normal/low IgG group. Lung function at baseline was significantly worse in adults with elevated IgG (median FEV1pp 54.3%, IQR: 42.4–70.0) compared to those with normal/low IgG (82.6%, IQR: 60.5–93.5, *p* = 0.001). Annualized FEV1pp decline was also greater in adults with elevated IgG (−1.2% per year, IQR: −2.7 to −0.6) than in those with normal/low IgG (−0.50% per year, IQR: −1.0 to +1.3, *p* = 0.006).

In this subgroup, 11 of 24 patients (45.8%) in the elevated IgG group met criteria for progression to severe lung disease, compared to 2 of 28 patients (7.1%) in the normal/low IgG group (*p* = 0.001). Although Pseudomonas aeruginosa was more frequent in adults with elevated IgG (75.0% vs. 53.6%, *p* = 0.11), this difference did not reach statistical significance (Table 1).

### 3.3. Severe Disease Group

Among the 14 patients who progressed to severe disease, one was younger than 18 years old at baseline, while the remaining 13 were adults. The median IgG Z-score in the severe disease group was 3.15 (IQR: 2.2–6.9), significantly higher than in the non-severe group (0.42, IQR: −0.59 to 1.85, *p* < 0.001). When comparing the severe disease group to the stable group, the annualized FEV1pp decline was greater in those who progressed to severe disease (−1.54% per year, IQR: −3.36 to −0.42) compared to those who remained stable (−0.76% per year, IQR: −2.37 to +0.70, *p* = 0.131), although this difference was not statistically significant.

Patients who progressed to severe disease were significantly older at their last follow-up compared to those who remained stable (median age: 27.6 years [IQR: 24.6–39.5] vs. 19.0 years [IQR: 12.0–32.0], *p* = 0.001). A higher proportion of female patients was observed in the severe disease group (68.8%) compared to the stable group (41.9%), a difference that reached statistical significance (*p* = 0.045). Although a greater proportion of patients with severe disease carried at least one severe CFTR mutation (75.0% vs. 51.4%), this difference did not reach statistical significance (*p* = 0.13). In the adult subgroup (>18 years old), the median IgG Z-score in those who progressed to severe disease was 2.95 (IQR: 2.25–5.50), significantly higher than in those who remained stable (0.80, IQR: 0.05–2.5, *p* = 0.002). The annualized FEV1pp decline in deteriorating adults was −2.1% per year (IQR: −3.99 to −0.46), compared to 0.77% per year (IQR: −1.3 to +0.70, *p* = 0.059) in the non-severe group, indicating a trend toward greater lung function decline in those who progressed.

Pseudomonas aeruginosa colonization was significantly more frequent in the severe disease group. Seven years prior to the last follow-up, 62.0% of patients who later deteriorated were already chronically infected, compared to 30.0% of those who remained stable. Four years before the last follow-up, Pseudomonas colonization rates increased to 75.0% in the severe disease group, compared to 37.3% in the stable group (*p* = 0.005).

### 3.4. Multivariable Analysis

**Figure 1:**
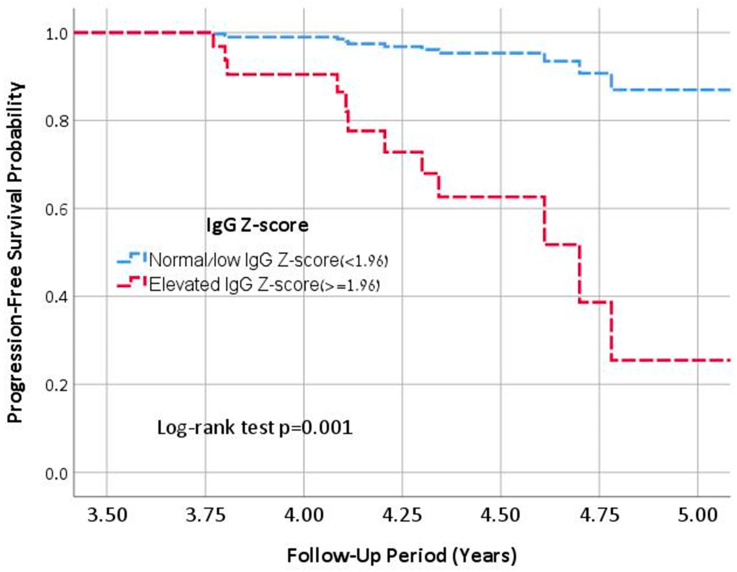
Kaplan-Meier Survival Analysis of Progression to Sever Lung Disease in PwCF with Elevated vs. Normal/Low IgG Levels.

In a multivariable Cox regression analysis, an elevated IgG was identified as an independent predictor of progression to severe lung disease (aHR: 9.1, 95% CI: 1.8–44.4, *p* < 0.001), after adjusting for annualized FEV1pp decline and Pseudomonas infection status (Figure 1). This association remained significant when the sensitivity analysis was restricted to patients aged ≥18 years (aHR: 11.3, 95% CI: 3.4–56.5, *p* = 0.008), adjusting for the same covariates.

## 4. Discussion

This study establishes elevated IgG as a significant predictor of progression to severe clinical lung disease in PwCF. Our findings align with previous studies showing an inverse correlation between IgG levels and lung function [5,6,7,8,9]. Proesmans et al. (2011) [7] reported that IgG Z-scores correlated inversely with FEV1pp in pediatric PwCF, with increasing IgG levels over time paralleling lung function decline. Similarly, Gur et al. (2021) found that higher IgG levels were associated with more severe disease parameters, including reduced spirometry values, increased lung clearance index (LCI), and more frequent antibiotic use [12]. While these studies primarily analyzed cross-sectional data or focused on laboratory IgG changes, our research establishes IgG as a longitudinal predictor of progression to severe lung disease in PwCF.

One of the most striking findings of our study is the high prevalence of severe lung disease among adults with elevated IgG levels. In the adult subgroup (>18 years old), nearly 46% of patients with elevated IgG progressed to severe lung disease, compared to only 7% of those with normal or low IgG levels. Notably, although baseline FEV1pp was significantly lower in the elevated IgG group, adults with elevated IgG also experienced a more rapid annual decline in lung function. This suggests that elevated IgG identifies a subgroup of PwCF at heightened risk for accelerated disease progression, independent of their initial lung function status.

The mechanistic link between elevated IgG and lung deterioration in CF remains incompletely understood but is likely driven by chronic inflammation [9,13,14].

Previous studies such as Moss et al. and Garside et al. have shown that elevated IgG levels in CF reflect persistent neutrophilic inflammation and chronic antigenic stimulation from airway infections, especially due to Pseudomonas aeruginosa colonization [13,14]. Consistent with these findings, chronic Pseudomonas colonization was significantly more frequent in our elevated IgG group (67.7%) compared to the normal/low IgG group (37.9%; *p* = 0.006).

Furthermore, several biomarkers have been associated with disease severity, including IL-8, TNF-α, and TGF-β1, which contribute to airway inflammation and remodeling [15,16,17]. However, these biomarkers are not routinely measured in clinical practice. In contrast, IgG is easily accessible and may serve as a practical surrogate marker of chronic inflammation in CF.

These findings suggest that elevated IgG may reflect an ongoing immune dysregulation and chronic inflammatory burden, which contribute to irreversible lung function decline in PwCF. Although IgG plays a protective role in opsonization and bacterial clearance, persistent elevation may suggest a maladaptive immune mechanism that contributes to airway remodeling and progressive lung function decline. In our study, PwCF with elevated IgG levels demonstrated worse clinical outcomes, including lower FEV1pp and increased association with progression to severe lung disease.

Recent studies, including a study by Pepe et al. [18], have demonstrated that initiation of highly effective CFTR modulator therapy, such as elexacaftor/tezacaftor/ivacaftor (ETI,) leads to significant reductions in serum IgG levels. Similar reductions were observed in IgA, γ-globulin, and leukocyte counts, likely reflecting reductions in airway inflammation and bacterial load. Further supporting these findings, a study by Schwarz et al. reported significant decreases in total serum IgG and IgE levels following ETI initiation, along with reduced proliferation of antigen-specific CD154⁺ T cells against common CF pathogens [19]. These results suggest that ETI may modulate adaptive immune responses, potentially through restoration of CFTR function in immune cells. However, the long-term prognostic utility of IgG in modulator-treated patients remains unclear. It is not yet known whether lower IgG levels in this context reflect sustained disease stability or represent transient suppression of systemic inflammation. Prospective, longitudinal studies in modulator-era cohorts are needed to clarify the clinical significance and temporal dynamics of IgG as a biomarker.

Until such data are available, routine IgG monitoring may continue to hold clinical value, particularly for patients who are not eligible for modulator therapy or those who exhibit suboptimal treatment response. Given its strong association with disease severity, chronic infection, and adverse outcomes, elevated IgG can aid in early risk stratification and inform key clinical decisions—such as the need for intensified therapy or timely referral for lung transplantation.

### Strengths and Limitations

A key strength of this study is its longitudinal design, which establishes IgG as a predictor of disease progression rather than just a severity marker. The inclusion of both pediatric and adult PwCF provides insight into IgG dynamics across different disease stages.

However, the small sample size limits statistical power. The timing of IgG elevation remains uncertain, making clinical interpretation challenging. Additionally, as a single-center study, findings may not be generalizable to CF populations with different treatment protocols. Data were collected prior to the widespread use of CFTR modulators.

## 5. Conclusions

This study builds upon established knowledge linking elevated serum IgG levels to lung function decline in PwCF. It demonstrates that elevated IgG (Z-score ≥ 1.96 SD) is associated with severe adverse clinical outcomes. Elevated IgG levels were correlated with older age, chronic Pseudomonas aeruginosa infection, and, among adults, lower baseline lung function and a more rapid decline in FEV1pp. An elevated IgG Z-score was a significant predictor of progression to severe lung disease over four years, even after adjusting for risk factors such as annual FEV1pp decline and chronic Pseudomonas colonization. These findings suggest that serum IgG levels reflect ongoing immune dysregulation and chronic inflammation in PwCF, which consequently impacts overall clinical status. Therefore, IgG serves as a meaningful and accessible prognostic biomarker for identifying high-risk individuals. Clinically, routine annual IgG monitoring could enhance risk stratification and facilitate earlier, more personalized interventions for patients at greatest risk of rapid disease progression.

## Figures and Tables

**Table 1:** Baseline characteristics and clinical features of patients with normal/low vs. elevated IgG levels—all PwCF and subgroup of 18 years and older.

Characteristics	All Patients			Subgroup—18 Years or Older		
	Normal/Low IgG (*n* = 66)	Elevated IgG (*n* = 31)	*p*-Value	Normal/Low IgG (*n* = 28)	Elevated IgG (*n* = 24)	*p*-Value
Age, years, median (IQR)	16.5 (9.8–26.3)	25.4 (18.2–36.0)	**0.004 ᵃ**	27.5 (22.3–38.0)	30.1 (22.4–37.8)	0.82
Follow-up time, years, median (IQR)	3.9 (3.7–4.2)	4.1 (3.8–4.4)	0.06	4.0 (3.7–4.4)	4.1 (3.8–4.4)	0.72
Female, *n* (%)	27 (40.9%)	17 (54.8%)	0.20	11 (39.3%)	15 (62.5%)	0.095
BMI Z-score, median (IQR)	0.04 (−0.78 to 0.56)	−0.03 (−0.89 to 0.90)	0.92	0.65 (0.05 to 1.18)	0.10 (−0.94 to 0.92)	0.052
Baseline FEV1pp, median (IQR)	96.0 (81.3–103.5)	64.0 (43.0–77.7)	0.06	82.6 (60.5–93.5)	54.3 (42.4–70.0)	**0.001 ᵃ**
Annual FEV1pp decline (%/year), median (IQR)	−0.64 (−2.07 to 0.74)	−1.18 (−3.3 to –0.42)	0.071	−0.50 (−1.0 to 1.3)	−1.2 (−2.7 to –0.6)	**0.006 ᵃ**
Pseudomonas colonization, *n* (%)	25 (37.9%)	21 (67.7%)	**0.006 ᵃ**	15 (53.6%)	18 (75.0%)	0.11
Severe CFTR genotype, *n* (%) ^b^	38 (57.6%)	16 (51.6%)	0.58	13 (46.4%)	14 (58.3%)	0.42
Pancreatic insufficiency, *n* (%)	42 (63.6%)	17 (54.8%)	0.41	14 (50.0%)	15 (62.5%)	0.37
CF-related diabetes, *n* (%)	11 (16.7%)	7 (22.6%)	0.49	9 (32.1%)	6 (25.0%)	0.57
Severe lung disease progression, *n* (%) ^c^	2 (3.0%)	12 (38.7%)	**<0.001 ᵃ**	2 (7.1%)	11 (45.8%)	**0.001 ᵃ**

Abbreviations: IQR—interquartile range; BMI—body mass index; FEV1pp—Forced Expiratory Volume in One Second Percent Predicted; CFTR—cystic fibrosis transmembrane conductance regulator. ^a^ Values in bold are statistically significant, *p*-value <  0.05. ^b^ Mutations I–III were classified as severe CFTR genotype mutations. ^c^ Defined as FEV1pp < 40%, referral for lung transplant, lung transplant, or death.

## Data Availability

The original contributions presented in this study are included in the article. Further inquiries can be directed to the corresponding author.

## Abbreviations

The following abbreviations are used in this manuscript:

CF	Cystic Fibrosis
PwCF	People with Cystic Fibrosis
CFTR	Cystic Fibrosis Transmembrane Conductance Regulator
IgG	Immunoglobulin G
FEV1pp	Forced Expiratory Volume in One Second Percent Predicted
BMI	Body Mass Index
IQR	Interquartile Range
HR	Hazard Ratio
CI	Confidence Interval
